# Human performance across decision making, selective attention, and working memory tasks: Experimental data and computer simulations

**DOI:** 10.1016/j.dib.2018.01.056

**Published:** 2018-02-21

**Authors:** Andrea Stocco, Brianna L. Yamasaki, Chantel S. Prat

**Affiliations:** Department of Psychology and Institute for Learning and Brain Sciences, University of Washington, United States

## Abstract

This article describes the data analyzed in the paper “Individual differences in the Simon effect are underpinned by differences in the competitive dynamics in the basal ganglia: An experimental verification and a computational model” (Stocco et al., 2017) [1]. The data includes behavioral results from participants performing three cognitive tasks (Probabilistic Stimulus Selection (Frank et al., 2004) [2], Simon task (Craft and Simon, 1970) [3], and Automated Operation Span (Unsworth et al., 2005) [4]), as well as simulationed traces generated by a computational neurocognitive model that accounts for individual variations in human performance across the tasks. The experimental data encompasses individual data files (in both preprocessed and native output format) as well as group-level summary files. The simulation data includes the entire model code, the results of a full-grid search of the model's parameter space, and the code used to partition the model space and parallelize the simulations. Finally, the repository includes the R scripts used to carry out the statistical analyses reported in the original paper.

**Specifications Table**

**Table:** 

Subject area	*Psychology*
More specific subject area	*Cognitive Psychology*
Type of data	*text files, log files, analysis scripts*
How data was acquired	*Human experiments conducted on a computer with the Eprime software (Psychological Software Tools, Pittsburgh, PA); Computer simulations*
Data format	*Raw data in binary format, analyzed data in Excel format, textual data*
Experimental factors	*Performance in three different experimental task (Simon task, Automated Operation Span task, Probabilistic Stimulus Selection task) and computer simulations*
Experimental features	*Correlations between difference facets of human performance across tasks; experimental predictions based on a simulated model of human behavior*
Data source location	*Seattle, WA, USA, 47.6553°N, 122.3035°W*
Data accessibility	*Data is available in this article, as well as on a public repository on the Cognition and Cortical Dynamics’ GitHub account:*https://github.com/UWCCDL/PSS_Simon

**Value of the data**

• The data includes individual human performance across three common experimental tasks that measure different cognitive abilities (decision-making, cognitive control, and working memory respectively).
• The ACT-R model code is available to inspect or integrate with other ACT-R models. The model provides an explanation for the existing correlation between two of the three tasks.
• Simulation data provides complete overview of model behavior across a large parameter space.
• R code is provided to ensure reproducibility of the experimental analysis published in the original paper [1].

## 1 Data

**Fig. 1:**
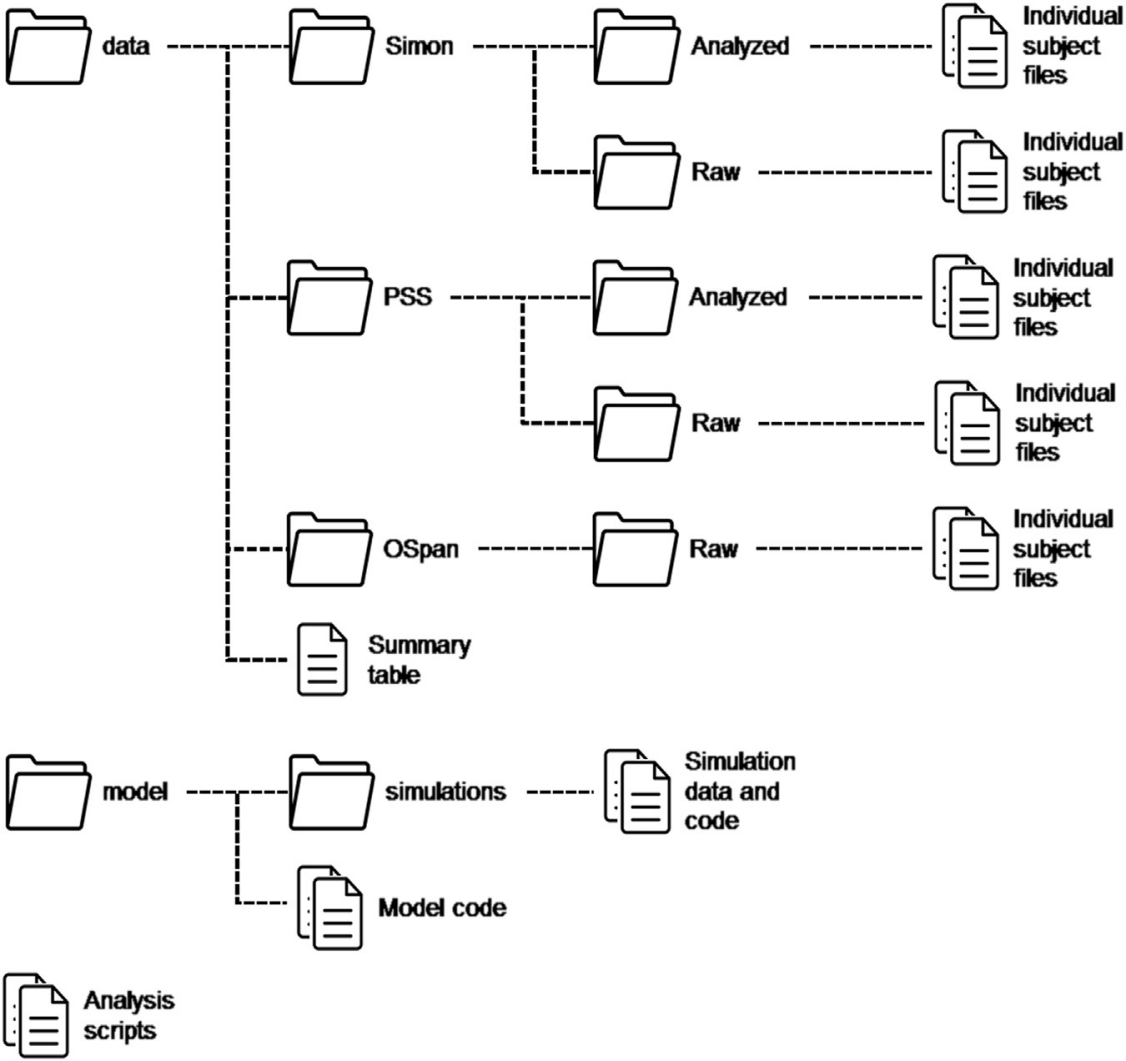
Organization of the complete dataset, as available on our laboratory's GitHub account: https://github.com/UWCCDL/PSS_Simon.

This paper describes the human experimental data and the computer simulations reported in the paper “Individual differences in the Simon effect are underpinned by differences in the competitive dynamics in the basal ganglia: An experimental verification and a computational model” [1]. In the case of experimental data, both group-level summary tables and individual datasets for each participant are available. Each individual dataset is available in both “raw” format (the output of the software used to present the experimental stimuli) and in “analyzed” format (the Excel worksheets used to compute individual performance measures). In addition to the experimental and simulation data, the analysis scripts used to compute the statistical results presented in the paper are also provided. The overall organization of the data follows the hierarchical structure shown in Fig. 1.

**Fig. 2:**
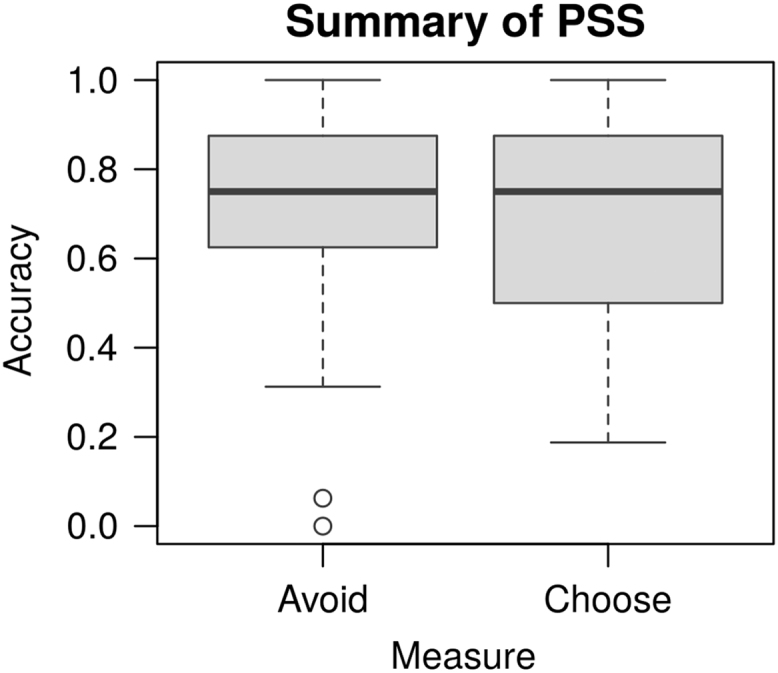
Tukey's boxplots of the mean Avoid and Choose accuracies in the Probabilistic Stimulus Selection (PSS) Task (Frank et al. [2]). The thick lines represent medians; the box represents the interquartile range; data points outside the range represent outliers.

**Fig. 3:**
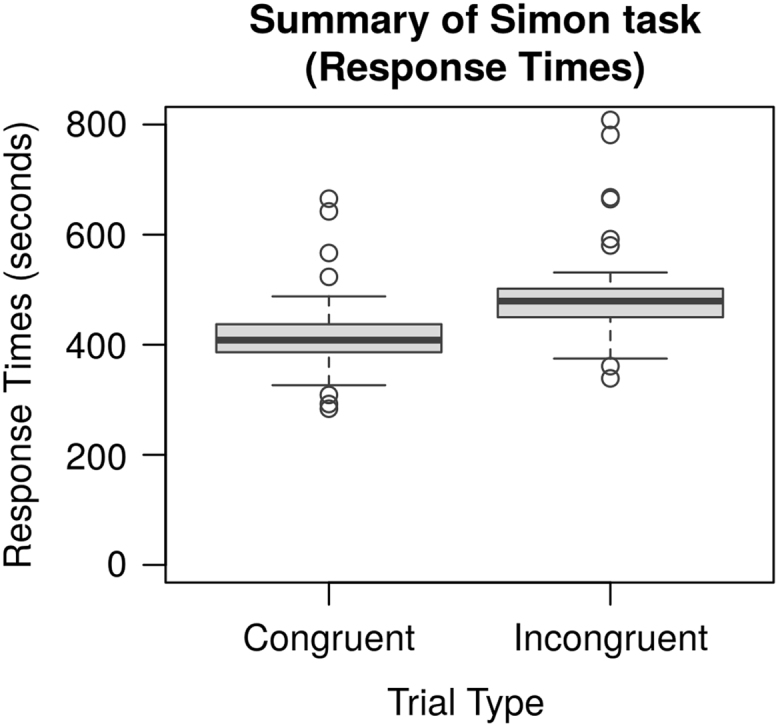
Tukey's boxplots of the mean response times for Congruent and Incongruent trials in the Simon task [6]. The thick lines represent medians; the box represents the interquartile range; data points outside the range represent outliers.

**Fig. 4:**
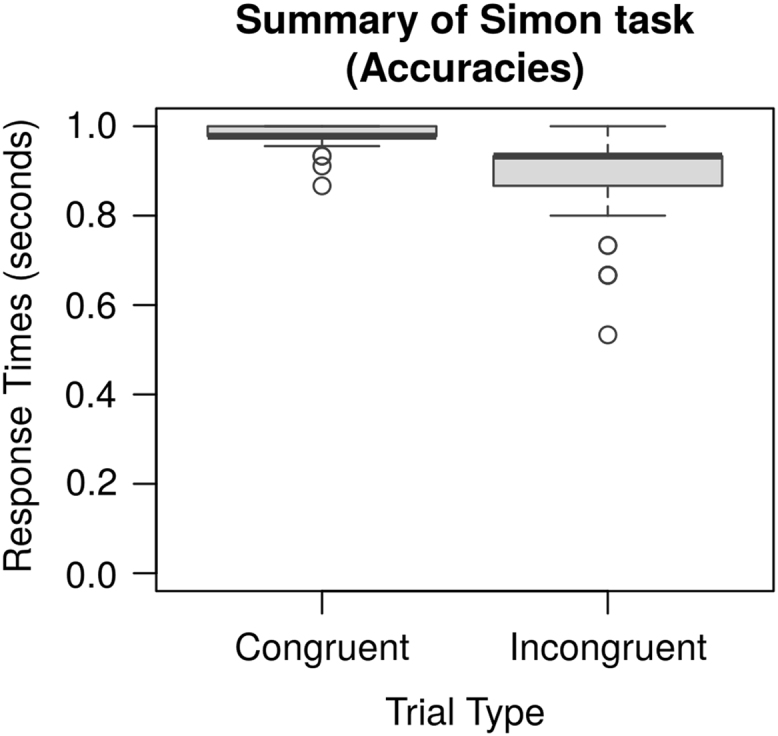
Tukey's boxplots of the mean accuracies (proportion of correct responses) for Congruent and Incongruent trials in the Simon task [6]. The thick lines represent medians; the box represents the interquartile range; data points outside the range represent outliers.

**Fig. 5:**
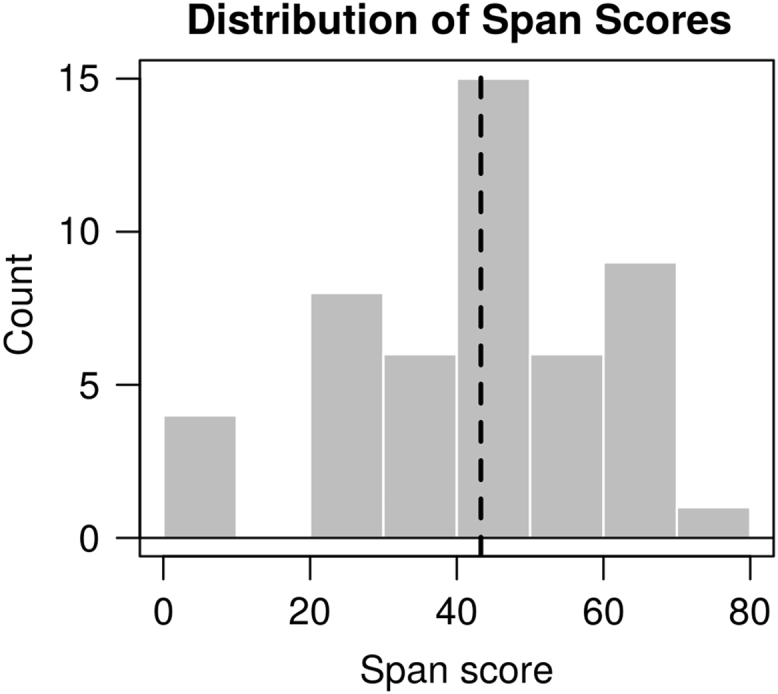
Histogram of the distribution of span scores in the Automated Operation Span task [4]. The dashed vertical line represents the group average.

All of the data reported in [1] are organized in two folders, named “data” and “model”. The “data” folder contains all of the experimental data. In three subfolders. Each of the subfolders contains the individual data obtained from each participant in the Simon [3], Probabilistic Stimulus Selection (PSS) [2], and Operation Span tasks [4], respectively. All the three subfolders contain a “Raw” directory, which holds the raw data generated by the Eprime software (Psychological Software Tools, Pittsburgh, PA). In addition, the “Simon” and “PSS” folders also have an “Analyzed” subfolder, which contains the data of individual participants in the format of a Microsoft Excel (Microsoft, Redmond, WA) workbook. Each workbook contains a sheet with the original data in table format, and one sheet with the summary results by conditions. A summary of the individual experimental results is provided in Table 1 (below). Figs. 2–4 provide a group-level overview of the main results in the PSS task (Fig. 2) and in the Simon task (Fig. 3, Fig. 4). Fig. 5 provides an overview of the distribution of the Operation span scores in our sample; the vertical line represents the sample mean.

**Table 1:** Summary of individual results across the three tasks.

**ID**	**PSS**			**Simon task**				**Operation Span**
				**Response times (in ms)**		**Accuracy**		
	**Choose accuracy**	**Avoid accuracy**	**Training trials**	**Incongruent**	**Congruent**	**Incongruent**	**Congruent**	**Span Score**
28020	0.750	0.875	180	580.286	484.814	0.933	0.956	25
28021	0.500	1.000	360	454.583	392.111	0.800	1.000	55
28022	0.563	0.750	120	429.769	408.318	0.867	0.978	48
28023	0.000	1.000	60	486.214	475.886	0.933	0.978	46
28024	0.500	0.438	360	667.786	484.546	0.933	0.978	50
28025	0.938	0.625	60	460.700	358.568	0.667	0.978	4
28026	0.875	0.688	60	453.267	404.721	1.000	0.956	36
28028	0.438	0.813	120	486.077	420.070	0.867	0.956	41
28029	0.688	0.750	360	486.929	428.909	0.933	0.978	0
28030	0.250	0.875	60	508.500	422.467	0.933	1.000	57
28031	0.875	0.938	240	490.571	476.933	0.933	1.000	43
28032	0.688	0.688	240	591.857	483.267	0.933	1.000	65
28033	0.688	0.625	180	426.818	363.023	0.733	0.978	62
28034	NA	NA	320	534.467	506.698	1.000	0.956	50
28035	1.000	0.688	120	473.467	425.773	1.000	0.978	61
28036	0.438	0.563	300	530.231	435.756	0.867	1.000	27
28037	0.500	0.563	120	492.533	437.227	1.000	0.978	6
28038	0.500	0.875	60	531.267	487.822	1.000	1.000	50
28039	0.875	0.438	60	470.357	401.756	0.933	1.000	43
28040	NA	NA	254	756.539	608.133	0.867	1.000	NA
28041	0.750	1.000	120	451.429	433.533	0.933	1.000	56
28042	0.438	0.563	60	449.929	388.818	0.933	0.978	68
28043	NA	Na	332	459.539	380.186	0.867	0.956	14
28044	0.750	0.813	180	379.400	292.341	0.667	0.978	23
28045	0.563	0.563	120	501.692	371.386	0.867	0.978	39
28047	NA	NA	205	524.143	457.455	0.933	0.978	15
28048	1.000	1.000	60	407.143	351.222	0.933	1.000	75
28049	0.813	0.938	60	492.929	426.977	0.933	0.956	46
28050	NA	NA	196	773.000	654.773	0.933	0.978	20
28051	1.000	1.000	180	361.083	326.477	0.800	0.978	55
28052	0.625	0.563	60	338.875	283.796	0.533	0.978	43
28053	0.750	0.938	120	501.786	463.796	0.933	0.978	35
28054	0.813	0.875	240	479.267	441.523	1.000	0.978	49
28055	0.813	0.750	60	523.385	400.930	0.867	0.956	43
28056	0.313	0.750	60	446.182	386.356	0.733	1.000	50
28057	0.688	0.750	120	447.500	390.556	0.800	1.000	6
28058	0.875	0.625	240	808.333	566.641	0.800	0.867	30
28059	NA	NA	247	531.200	472.778	1.000	1.000	62
28060	0.813	1.000	120	466.077	424.067	0.867	1.000	64
28061	0.688	0.750	360	487.214	421.556	0.933	1.000	29
28062	0.375	0.313	120	475.071	372.886	0.933	0.978	63
28063	1.000	0.938	60	413.083	367.977	0.800	0.956	68
28064	NA	NA	292	574.867	726.415	1.000	0.911	19
28065	0.625	0.875	60	374.857	337.854	0.933	0.911	46
28066	NA	NA	296	402.455	351.977	0.733	0.978	47
28067	0.375	0.000	120	504.786	401.222	0.933	1.000	39
28068	1.000	0.063	120	781.067	665.357	1.000	0.933	62
28069	0.875	0.875	60	503.429	420.409	0.933	0.978	32
28070	1.000	0.750	360	489.357	523.233	0.933	0.956	69
28071	0.188	1.000	60	488.308	402.046	0.867	0.978	60
28072	0.563	0.938	60	494.933	476.477	1.000	0.978	42
28073	0.938	0.563	120	664.500	642.200	0.933	1.000	48
28074	0.875	0.750	180	394.300	308.796	0.667	0.978	42
28075	0.313	0.938	120	454.583	392.111	0.800	1.000	55
28076	1.000	0.750	60	476.231	405.976	0.867	0.933	23
28077	0.750	0.688	360	499.923	397.356	0.867	1.000	24
28078	0.875	0.750	180	427.077	375.591	0.867	0.978	27
28079	0.938	0.875	60	476.800	420.186	1.000	0.956	37

The “model” folder contains the model code and a “simulations” subfolder. The “simulations” subfolder contains the text log files of the simulations (“simulations.txt”) as well as the Python code used to generate the simulations.

Finally, the base folder also contains two scripts used to analyze the experimental and simulation results of the [1] paper. Both scripts consist of code in the R programming language. The “analysis.R” script contains the code used to generate the statistical results reported in the paper, as well as its plots and figures. The “model-flexibility-analysis.R” scripts contains additional code for the analysis of the model's performance using the methods described in [5]. The results of these analyses were eventually omitted from final publication, but can be examined by running the file's code. A copy of this repository is also available on the Cognition and Cortical Dynamics’ GitHub account page, at https://github.com/UWCCDL/PSS_Simon.

## 2 Materials and methods

### 2.1 Participants

Fifty-eight healthy individuals were recruited for the experiment (age = 18–34 years, 44 females). Data from 8 participants (5 female) were not analyzed due to an inability to attain learning criteria required during the PSS task learning phase. Subject numbers for unusable participants are included in analysis scripts. All participants were recruited from the student population of the University of Washington campus and the surrounding Seattle area and received monetary compensation in exchange for their time. All participants provided written informed consent in accordance with the ethical guidelines established by the IRB prior to the start of the experiment.

### 2.2 Experimental procedures

The PSS task [2], the Simon task [3], and one test of complex working memory span (the Operation Span task [4]) were administered to participants as part of a larger cognitive battery assessing cognitive capabilities in participants who went on to participate in a training experiment not reported herein. With the exception of the three tasks described herein, no other experimental task was selected for this specific analysis, and no dataset was discarded after having been considered for this study. All of the tasks were performed on a computer, in front of a 21” LCD screen, using a standard keyboard to respond. Stimulus presentation and response collection were controlled through the E-prime software (Psychological Software Tools, Pittsburgh, PA).

#### 2.2.1 The Simon task

The “Simon” sub-folder contains the data from the Simon task [3], [6], a response interference task used to collect individual measures of cognitive control. During the task, participants were presented with one of two shapes, either a black square or a black circle, on a white background. Participants were instructed to respond to one shape (e.g., squares) with their right hand, and to the other shape (e.g., circles) with their left hand. Each trial was introduced by an 800 ms fixation, followed by a 250 ms time delay, followed by a stimulus (circle or square) that remains on the screen for either 3000 ms or until a response was recorded. Trials were either congruent (e.g., a stimulus associated with a left response and presented on the left half of the screen) or incongruent (e.g., a stimulus associated with a left response and presented on the right half of the screen), with congruent trials making up 75% of the total count. The task consisted of 64 trials divided into 4 blocks of 16 trials each.

#### 2.2.2 The Probabilistic Stimulus Selection (PSS) task

The “PSS” sub-folder contains the data from Probabilistic Stimulus Selection (PSS) task, an iterative decision-making task that is used to collect measures of basal ganglia function [2]. The task consists of two consecutive phases, a training and a test phase. In both phases, participants performed multiple decision-making trials in which they were asked to choose one of two Japanese Hiragana characters, placed at the left and right side of the screen. Participants indicate their response by pressing the keys “1” (for the left character) or “0” (for the right character) on a standard keyboard. A total of six stimuli are presented, each of which is associated with a unique probability of success. During the training phase, the six stimuli are presented in three fixed pairs. To ensure that participants can discriminate the relative success probability for each stimulus, the training phase is repeated until each participant's accuracy has reached a predetermined criterion. After a maximum of six repetitions of the training phase, participants move on to the test phase. The test phase presents each of the 15 possible combinations of stimuli four times, for a total of 60 trials. The decisions made during the test phase yield two distinct measures of performance: the accuracy in choosing the most rewarding stimulus against all the others (*Choose accuracy*, i.e., the proportion of choices in which stimulus A is preferred over C, D, E and F) and the accuracy in avoiding the least rewarding stimulus when it is paired with all the other (*Avoid accuracy*, or the proportion of choices in which stimuli C, D, E, and F were preferred over B).

#### 2.2.3 The Operation Span task

The “OpSpan” folder contains data from the automated version of the Operation Span task [4], a non-verbal test of complex working memory span. In the Operation Span task, participants memorize a sequence of letters (e.g., “L”, “Q”, and “S”) that are presented in alternation to the evaluation of arithmetic expressions (e.g. “(2 × 5) − 9 = 2”). While memorizing the letters, participants also have to indicate whether the expression was mathematically true or false. After the presentation of the last letter, participants indicate all the letters that had been presenting since the beginning of the trial by selecting them in the order in which they appeared from an array of 4 × 3 letters. The number of letters to be memorized varies pseudo-randomly across trials, up to a maximum number of seven [4]. Working memory capacity was measured in terms of the *Span Score* [4], which ranges from a minimum of 0 to a maximum of 75, the latter indicating that all of the trials were correctly remembered.

### 2.3 Computer simulations

The computer simulations were obtained from a computational neurocognitive model based on the ACT-R cognitive architecture [7], version 7.4, and implemented in Common Lisp. All of the model code and simulations are included in the “model” subfolder of the data repository.

#### 2.3.1 Model code files

The “model” subfolder contains the entire model code, divided into three files. The “simon-model.lisp” file contains the main ACT-R model code. The “simon-device.lisp” file contains the code necessary to present the Simon task to the model and collect the model responses. Specifically, this file contains the data structures necessary to implement an ACT-R “device”, that is, a Common Lisp object that can provide inputs to, and receives responses from, an ACT-R model. Finally, the “simon-simulations.lisp” file contains the code to execute multiple runs of the model under different parameters and conditions.

#### 2.3.2 Model simulations

The “simulations” subfolder contains the results of a full grid-search simulation of the model's behavior across five different parameters (see [1] for details). All of the simulation results are contained in a single text file, “simulations.txt”. Each row of the file represents the average performance of the model over 100 runs under a specific combinations of parameter values. The “gen-simulations.py” file contains the Python code that was used to set up the simulations. The Python code generates over 100 Lisp files, each of which is parametrized to examine a different portion of the complete parameter space. The use of multiple Lisp files permits to run the simulations in parallel on multi-core computers.
