# Supplementary material for: Human performance across decision making, selective attention, and working memory tasks: Experimental data and computer simulations
Source: Data Brief. 2018 Feb 21;17:907–14. doi: 10.1016/j.dib.2018.01.056 (PMC5988376; doi:10.1016/j.dib.2018.01.056)
Supplement: Supplementary file 1 — Transparency document [file mmc1.doc]

*Conflict of Interest Form*

Conflict of Interst: None
